# Sex Differences in Mechanisms and Outcome of Neonatal Hypoxia-Ischemia in Rodent Models: Implications for Sex-Specific Neuroprotection in Clinical Neonatal Practice

**DOI:** 10.1155/2012/867531

**Published:** 2012-02-14

**Authors:** Courtney A. Hill, R. Holly Fitch

**Affiliations:** Department of Psychology, University of Connecticut, 406 Babbidge Road, Storrs, CT 06269, USA

## Abstract

Clinical findings show that male infants with hypoxic-ischemic injury (HI) fare more poorly than matched females on cognitive outcomes. Rodent models of neonatal hypoxia-ischemia support this difference, with data showing that perinatal brain injury leads to long-term behavioral deficits primarily in male rodents and in female rodents treated with early androgens. Results support the idea that sex-specific gonadal hormones may modulate developmental response to injury and dovetail with overwhelming evidence of developmental androgen effects on typical brain morphology and behavior. However, mechanisms underlying sex differences in response to early brain injury may be more complicated. Specifically, activation of cell death pathways in response to HI may also differ by sex. In females, the preferential activation of the caspase-dependent apoptotic pathway may actually afford greater protection, potentially due to the actions of X-linked inhibitor of apoptosis (XIAP) within this pathway. This contrasts the pattern of preferential activation of the caspase-independent pathway in males. While an integrated model of sex-specific hormonal and genetic modulation of response to early injury remains to be fully elucidated, these findings suggest that infants might benefit from sex-specific neuroprotection following HI injury.

## 1. Introduction

Perinatal hypoxic-ischemic injury (HI; concurrent oxygen/blood deprivation in the brain) represents a major cause of mortality and long-term neurologic morbidity in premature/very low-birth-weight (VLBW) infants (<1500 g) and in term infants suffering birth trauma [1]. In premature infants, the vulnerability of the underdeveloped neural vascular system, coupled with poor cerebral autoregulation [2], can often result in intraventricular or periventricular hemorrhagic injury (IVH-PVH; bleeding within or surrounding the ventricles [3]). These bleeds, primarily located in the subependymal germinal matrix, lead to some immediate cell necrosis as well as a progressive apoptotic cell death cascade of germinal matrix and glial precursor cells [3]. In addition, poor cerebral autoregulation (including reperfusion failure) in preterm infants can lead to periventricular leukomalacia (PVL), a nonhemorrhagic ischemic injury associated with loss of white matter surrounding the ventricles [4]. Moreover, underdevelopment of the lungs can lead to reduced oxygenation of the blood, thus resulting in hypoxic conditions within the brain of premature infants. Animal models of acute preterm HI injury include the Rice-Vannucci model of unilateral carotid artery ligation followed by a period of exposure to 8% oxygen prior to postnatal day 7 (P7), typically performed in rodents [5–7]. Additional models are also used in which fetal blood supply is diminished by clamping of the placental blood supply [8, 9], and/or by raising dams in a low-oxygen environment for a period of days [10, 11].

 In term infants, HI injury typically results from complications of birth (e.g., cord compression, placental disruption/failure, or cord asphyxia [1, 7, 12–14]) and can result in cerebral white matter injury typical of cerebral palsy [15] or in gray matter injury [1, 16, 17]. Thus, term-born children experiencing asphyxia exhibit injuries more typical of hypoxic-ischemic encephalopathy (HIE), in contrast to injuries exhibited by preterm children (i.e., IVH-PVH, PVL). Animal models of term injury include the Rice-Vannucci method performed on P7-P10 [5–7] or methods of middle cerebral artery occlusion (MCAO, [18]). Although the mechanisms of neural damage in preterm versus term populations differ, these varied forms of injury result in similar activation of acute necrotic and delayed apoptotic cell death mechanisms that impact on cell populations most vulnerable during the time of injury [5–7].

Not surprisingly, the long-term consequences of neonatal HI injury in both populations can be severe. Nearly 50% of term-born infants suffering severe HIE die within weeks of birth, while up to 25% of those surviving exhibit permanent neuropsychological dysfunction [7]. Similarly, a 50% mortality rate exists for preterm infants experiencing severe HI, with 80% of survivors experiencing long-term complications [19], including reductions in cerebellar [20], cortical, and hippocampal volumes [21] associated in turn with cognitive and behavioral deficits, deficits in verbal and language domains [22, 23], reduced IQ measures [24], cerebral palsy, and mental retardation [25]. What is surprising, however, is the disproportionate incidence of, and increased severity of effects following, neonatal HI injury in males. Not only are male infants more vulnerable to perinatal insult (showing higher incidence of IVH and increased rates of mortality from prematurity or stillbirth), they also suffer more long-term cognitive deficits as compared to females with comparable injury [23, 25–31]. In fact, males in general show increased risk for brain-based developmental disorders, including speech and language disorders, stutter, dyslexia, autism, learning disabilities, attention-deficit-hyperactivity disorder, and cerebral palsy as compared to females [26, 27, 30, 32]. Importantly, males suffering intracranial bleeds at birth also display significantly lower full-scale, verbal, and performance IQ at early school age as compared to females matched for degree of prematurity and severity of intracranial bleed [33]. Overall, evidence suggests that among infants at risk for HI, females may be at a quite significant advantage as compared to their male counterparts who are two times more likely to experience prenatal anoxia, hemorrhage, and infection, and 1.8 times more likely to suffer cerebral birth trauma [26–28, 30].

Despite the overwhelming evidence of sex differences in outcome following neonatal HI injury, many researchers appear to remain naïve to the importance of sex in perinatal injury models and continue to utilize only male animals in research studies. However, recent work concerning hormones present during the perinatal period, as well as sex differences in mechanisms of cell death, have begun to illustrate dynamic and differing processes occurring in the neonatal brain following injury and emphasize the need for studies to include both sexes. This work suggests, first, that the substantially elevated level of testosterone present in human male fetuses during gestation through the first year of life [34–36] may enhance neuronal excitotoxicity following hypoxic-ischemic insult [37, 38] and may contribute to exacerbated deficits in males [39, 40]. Second, evidence suggests that following such injury, male and female cells diverge in the proportional activation of caspase-dependent and caspase-independent pathways leading to apoptotic death [41–43]. In fact, this difference may contribute to outcomes that show males to be more vulnerable to early brain damage [44–46]. Finally, data indicate that females may possess a gene-linked advantage through a family of inhibitors of apoptosis (IAPs [47]), the most potent being X-linked IAP (XIAP [48]). XIAP is known to act on the caspase-dependent apoptotic pathway [48–51], and it is possible that increased expression of XIAP [52] in females may contribute to a female advantage following neonatal HI. Taken together, this evidence suggests an interplay of hormonal modulation and genetically determined apoptotic mechanisms, through which perinatal females may be afforded a level of protection against HI injury that is greater than for perinatal males. The current paper will focus on factors that may play key roles in the outcome of hypoxic-ischemic events experienced by males and females, including perinatal exposure to sex-specific gonadal hormones and sex-specific cell death mechanisms. Research in this area could lead to the discovery and clinical implementation of sex-specific neuroprotectants for infants suffering from HI injury.

## 2. Early Hormonal Factors

Sex differences in androgen levels represent one principal difference in the male versus female neonatal brain and lead to substantial effects on brain morphology and subsequent behavior [34–36, 53–59]. Human testes develop around gestational week (GW) 6, with testosterone from the testes—as well as from the fetal adrenals (as a by-product of corticosteroid production)—circulating at detectable plasma levels in males by GW 8 [34, 35]. Testosterone secretion, however, is highest from GW 10 to 20, falling to lower levels by GW 24, followed by a second transient testosterone surge on the day of birth (in response to the drop in placental estrogen). In humans, testosterone levels gradually increase during the first week of life and remain high for the first year, peaking during the 3rd-4th month at levels similar to the second stage of puberty (200–300 ng/dL; [35]). Through aromatization, testosterone can be converted to 17-B estradiol, thus allowing it to bind to estrogen receptors within the brain [53, 54, 60]. In fetal male rats, where plasma testosterone is significantly higher than female littermates beginning at embryonic day 18 (E18) through P5 [61], the conversion of circulating testosterone to estradiol results in neural and behavioral masculinizing effects [36, 53]. However, no studies of which we are aware directly support this mechanism in humans. Difficulties in ascertaining the role of aromatization in human sexual differentiation reflect experimental constraints [53], but some evidence does support a role for aromatization in human development [62].

Human female fetuses are also exposed to androgens from the fetal adrenal glands, as well as the maternal adrenals, ovaries, and fat—though the amount is insufficient for masculinization. In humans, it is believed that a negative feedback loop between the fetal adrenal cortex and the anterior pituitary corticotrophins minimizes female adrenal androgen secretion, acting as a transient mechanism that safeguards early human female development from virilization [63]. The fetal ovaries also develop at approximately GW 7, though no circulatory estrogen of fetal ovarian origin is present until very late in gestation [34, 35]. Likewise, studies of ovarian secretion of estrogen in the rat reveal detectable levels 5 days after birth (corresponding to the late third trimester human; [56]), although some central steroidogenesis may occur [64]. In rodents, females circumvent masculine development via maternal estrogen (consistent with masculinization via intracellular conversion of androgens as discussed above) through alpha-fetoprotein, a binding globulin found in late-gestation fetuses. This protein binds to estrogen within the bloodstream thereby rendering it inactive and preventing virilization of the female rodent brain [36, 54].

### 2.1. Testosterone and Brain Injury

As noted above, the presence of testosterone during development represents one of the foremost differences between neonatal male and female brains. However, despite a large and dynamic literature concerning modulatory effects of gonadal hormones on pathologic and behavioral response to stroke injury in adults, research data concerning hormonal modulation of injury in neonates remains scant (but see [65]). A brief review of this adult literature illustrates that studies using an induced injury model simulating adult stroke—middle cerebral artery occlusion (MCAO)—show consistent evidence that males benefit after injury from acute testosterone depletion, while the presence of testosterone increases glutamate toxicity following injury [37]. Furthermore, testosterone and its metabolite dihydrotestosterone (DHT) have been shown to increase stroke damage in young adult rats [37, 66, 67], while testosterone concentration was found to be inversely associated with stroke severity and 6-month mortality [68, 69]. Interestingly, young male rats were also found to incur larger strokes than their older counterparts, an effect hypothesized to be due to the ability of testosterone to alter the susceptibility of the brain to ischemic damage in an age-dependent manner. Alternately, though aromatase levels are stable over age (and thus the protection afforded to aged males is not likely due to increased capability for aromatization of testosterone to estrogen [70]), the declining effects of stroke damage in older males may reflect higher levels of testosterone in young rats [70].

 In neonatal animals, baseline sex differences have been seen with an early hypoxia model [10, 71] as well as an HI model [40, 72, 73] of brain injury—both of which have shown that males exhibit increased brain volume loss [10, 40, 71, 74], disrupted myelination [10], and increased behavioral deficits [40, 71–73] following injury as compared to like-treated females. As with adult injury models, there is also some evidence that the presence of androgens can exacerbate induced brain damage—for example, following GABA-A mediated excitotoxicity [65]. Other studies of a rat model of focal ischemic injury leading to developmental cortical malformation (microgyria) found that androgenizing female rat pups via testosterone propionate (TP) prior to and following induction of microgyria via focal cortical freezing lesion on P1 led to a developmental shift in medial geniculate nucleus (MGN) neuronal size distribution in adulthood—similar to that seen in male microgyric rats—while vehicle-treated microgyric females were found to be identical to sham females (and showed no disruption of the MGN [39]). With regard to the specific long-term effects of testosterone as a modulator of neonatal HI injury, we are aware of only one study performed to date [40]. In this study, vehicle-treated male and female rat pups, as well as female rat pups that had been treated on postnatal days 1–5 (P1–5) with superphysiologic levels of testosterone propionate (TP), received the Rice-Vannucci HI procedure [5] on P7. Results showed subsequent deficits in auditory processing ability in males and TP-treated females with induced neonatal HI, while no effect of HI was found in vehicle-treated females [40], thus demonstrating the apparent deleterious effects of androgen exposure in modulating behavioral deficits associated with HI.

From these cumulative studies, it is evident that testosterone acts in some manner to exacerbate the response to early hypoxic-ischemic injury in rats, though the specific mechanism(s) of action remain to be defined. Moreover, since an aromatizable form of testosterone propionate was used in the above study [40], it is not possible to determine whether these effects were modulated directly by androgens or via intracellular conversion to estrogen. Future studies looking at neonatal HI while manipulating testosterone receptors, estrogen receptors, and/or aromatase blockers could potentially dissociate or clarify this issue. Nonetheless, further research in the area of neonatal testosterone exposure may help to better characterize the protection afforded to females, which potentially could be adapted to males through some form of neuroprotective treatment. Although the generalized use of androgen-blocking manipulations in male infants would be a clinically untenable intervention, a viable option could be to identify the delineated mechanisms of androgenic exacerbation of injury and block those specific effects only.

### 2.2. Estrogen and Brain Injury

It must also be noted that evidence from animal models of adult stroke shows substantial beneficial and protective effects of estrogen modulation [75–77]. In fact, adult female animals have a lower incidence of naturally occurring stroke [78] and show less sensitivity than male animals to the damaging effects of focal or global ischemic injury [79], and—in strains displaying conditions known to be stroke risk factors in humans (i.e., hypertension)—female animals display less tissue damage than males following induced stroke [80]. This female advantage has been attributed at least in part to protective effects of ovarian steroid hormones, since interventions that reduce estrogens (i.e., ovariectomy, estrogen receptor blockade, and natural aging) have all reduced differences in stroke outcome between the sexes [75]. Further, induced stroke during metestrus (when estrogen is lowest) increases tissue damage in comparison to strokes occurring during proestrus (when estrogen is highest; [77]). It should be noted that the effects of estrogen on adult human stroke are less clear, and many of the successful studies of estrogen replacement in animal models have failed to translate to human clinical populations [75, 76]. Nonetheless, whatever the protective mechanism(s) of estrogen may be, they are less likely to fully account for the neonatal effects described here, since female protection has been shown in animal models of neonatal brain injury, when minimal circulating estrogen from the quiescent neonatal ovaries is present [40, 68, 72, 73, 81, 82] although central steroidogenesis may occur [64]. Still, it is certainly possible that some late developmental beneficial effects of ovarian estrogen on neural reorganization after injury could occur.

 These hormonal data, taken together, suggest that early androgen exposure in males may be a primary contributor to the modulation of sex differences in response to HI injury (although whether these effects occur through aromatization cannot be determined, based on data collected to date). However, another promising line of research aimed at exploring sex differences in response to brain injury has begun to examine possible sex differences in the mechanisms of cell death following injury. These findings have led researchers to believe that hormonal differences are not the only key factor modulating sex differences in response to injury, and that the apoptotic cascade may be differentially activated in the male and female brain following HI.

## 3. The Apoptotic Cascade

**Figure 1:**
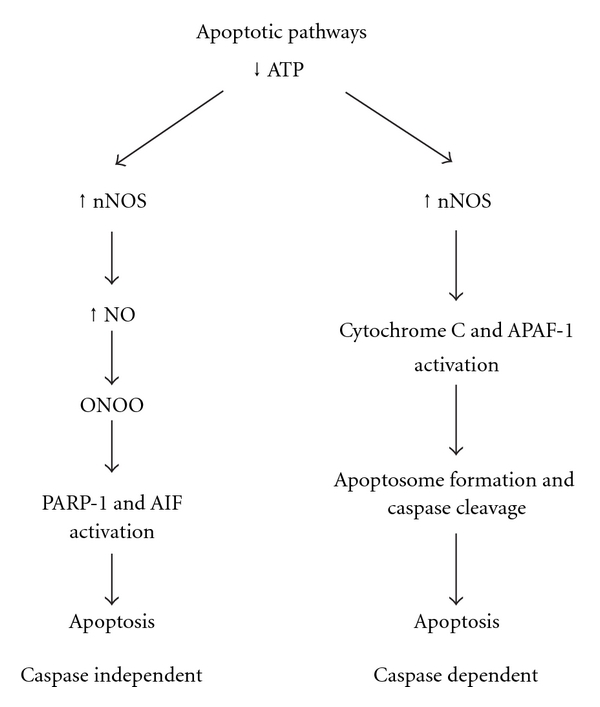
A diagram of the progression of caspase-independent and -dependent apoptotic mechanisms.

Apoptosis, or programmed cell death, can be triggered by various events including DNA damage, cytotoxic drugs, a lack of survival signals, and developmental death signals—among a variety of other mechanisms [83]. With regard to neuronal death in response to hypoxia-ischemia, events are initially triggered by a deprivation of oxygen and glucose supply to the cell(s), which depresses both adenosine triphosphate (ATP) synthesis as well as the cellular uptake of glutamate. The accumulation of excess extracellular glutamate triggers an increase in glutamate receptor (NMDA, AMPA, and kainate) activation and prolonged depolarization, leading to increased calcium and sodium influx. Sodium influx through AMPA and kainate receptors leads to cell swelling and rapid necrotic cell death, while calcium influx through NMDA and AMPA receptors lacking the GluR2 subunit (rendering the channel open to calcium) activates neuronal nitric oxide synthase (nNos). nNos, in turn, leads to the production of the free radicals, nitric oxide (NO), and peroxynitrate (ONOO). In caspase-*independent*-mediated cell death, a reduction of nicotinamide adenine dinucleotide (NAD^+^, a high energy molecule) is caused by activation of poly(ADP-ribose) polymerase-1 (Parp-1, a DNA repair enzyme), leading to release of apoptosis-inducing factor (AIF) and endonuclease G from the mitochondria to the nucleus of the cell, and ultimately cell death [49–51]. Through a second caspase-*dependent* pathway, the increase in nNos ultimately leads to mitochondrial dysfunction and the translocation of cytochrome-c from the mitochondria to the nucleus, signaling apoptotic protease-activating factor-1 (APAF-1) and the formation of the apoptosome. The apoptosome binds with caspase-9 (the initiator caspase), which in turn cleaves downstream caspases 3, 6, and 7 (effector caspases) causing chromatin condensation, DNA fragmentation, and ultimately cell death [42, 50, 83–85], (see Figure 1).

Interestingly, research has revealed the sexes to differentially favor one of these two pathways (though not exclusively), with females relying more heavily on the caspase-dependent pathway and males largely utilizing the caspase-independent pathway of cell death following HI insult. These data derive from injury models in both adult stroke (MCAO) and neonatal HI models [41–46, 80, 86, 87].

### 3.1. Apoptotic Cascades and Brain Injury

In adult stroke models, male and female Parp-1-deficient mice both display a reduction in Parp-1 and AIF production, suggesting the activation of the caspase-independent apoptotic pathway by both sexes. However, only male Parp-1-deficient animals exhibited a reduction in stroke-induced brain damage following MCAO [86]. Likewise, female (but not male) mice were largely resistant to endotoxin-induced mortality, and Parp-1 inhibition decreased endotoxin-induced vascular and inflammatory response in male (but not female) mice [88]. Interestingly, ovariectomy partially reversed the protection normally seen in females, suggesting a modulating role of estrogen [88]. Similarly, inhibition of Parp-1 and nNOS was found to protect male animals from the damaging effects of MCAO (but not females, [89]). In fact, Parp-1 inhibition *increased* stroke damage in intact females and estrogen-replaced ovariectomized females, again suggesting a mediating role of estrogen [89]. Though the results of these two studies suggest that ovarian hormones may play a role in modulating caspase-independent cell death in adult models, it is important to note that sex differences in neonatal HI are found when minimal circulating estrogen is present (though evidence indicates central steroidogenesis may be occurring [64]). These neonatal studies also report evidence of predominant male use of the caspase-independent pathway, suggesting such sex differences may not be exclusively mediated by exogenous hormones (see below). Likewise, preferential activation of the caspase-dependent apoptotic pathway has been seen in adult female animals following MCAO, as increased cytochrome-c release and caspase-3 cleavage was found relative to males, while inhibition of caspase activation was found to be neuroprotective in female animals only. This benefit was extended to ovariectomized and estrogen-replaced female animals, indicating these effects to be independent of hormones [80].

 Recent work exploring cell death mechanisms in neonatal models also indicates sex differences in the preferred apoptotic pathway following early HI [41, 42]. With regard to the caspase-independent apoptotic pathway, both Parp-1 and AIF have been found in higher concentration in the brains of male mice following P9 HI injury, as compared to the brains of HI-injured females [43]. Likewise, significant protection from P7 HI injury has been shown in Parp-1 knockout male mice, though no comparable protection was seen in females [44]. Other studies report that cytochrome-c and various caspases—which are active in the caspase-dependent pathway—have been found in higher concentration in female as compared to male mice following P9 HI injury [43]. Likewise, inhibition of caspase cleavage has been shown to be neuroprotective in female (but not male) animals following P3 or P7 HI [45, 46], where translocation of cytochrome-c was prevented. Finally, neuronal cultures (absent of circulating hormones) subjected to cytotoxic challenge showed differing pathways of cell death—with XY neurons predominantly utilizing the AIF-mediated caspase-independent apoptotic pathway and XX neurons activating the cytochrome-c, caspase-dependent apoptotic pathway [38]. It should be noted that no studies (of which we are aware) have quantified the exact proportional activation of caspase-dependent and -independent apoptosis for either sex, but instead have measured the activation of elements specific to each pathway or the relative protection afforded to one sex over the other following knockout/inhibition of elements specific to one pathway as stated above. These sex differences are found to be significant in magnitude.

From these studies, it is evident that male and female neurons undergoing apoptosis capitalize on pathways that show sex differences unlikely to be exclusively related to hormones (though these apoptotic pathways are not exclusive to sex). However, detailed assessment of the potential interaction between the early (or concurrent) presence of gonadal hormones, and the activation of sex-specific apoptotic cascades, remains to be defined. Nonetheless, apoptosis is a major contributor to neuronal cell death and tissue loss following neonatal HI, and the development of neuroprotectants aimed at targeting the mechanisms most utilized by each sex represents a valuable venue of investigation for therapeutic interventions.

## 4. A Gene-Linked Female Advantage

An alternative or additional explanation for sex differences seen in neonatal HI outcome involves endogenous inhibitors of apoptosis. During development, the apoptotic cascade is a highly regulated process critical for healthy development and maintenance of tissue. This process of programmed cell death is counterbalanced by antiapoptotic signals that promote the survival of cells. A family of proteins, known as inhibitors of apoptosis (IAPs), serve as endogenous inhibitors of cell death [47, 90] and have been found to regulate apoptosis by blocking both the intrinsic and extrinsic mechanisms. Specifically, IAPs directly bind to and inhibit initiator and effector caspases [47, 49, 51]. The function of IAPs has recently been extended beyond its initial role in development and is now thought to play a role in processes such as cancer, tumor formation, autoimmune diseases, neurodegenerative disorders, and most recently, cell death following brain injury [51, 91].

Of the known IAPs, X-linked IAP (XIAP) is recognized to be the most potent [48]. XIAP effectively binds to the initiator caspase (caspase-9) and halts further cleavage of downstream caspases (caspases 3 and 7), thus preventing cell death [48–51]. XIAP has also been shown to bind and inhibit caspases 3 and 7 directly [48], and *in vitro* studies have revealed XIAP to severely inhibit nuclear destruction and cytochrome-c-induced caspase activation [48]. XIAP expression has been confirmed in both rodent and human brains following ischemic injury [92]. Moreover, it is understood that genetic balancing in females occurs via random inactivation of the second X chromosome; however, 15% of genes located on the second X chromosome always escape inactivation, and an additional 10% sometimes escape inactivation [52]. Therefore, it is possible that females present with an increased expression of XIAP relative to males. And since XIAP acts specifically on the caspase-dependent pathway of cell death preferentially activated in females, XIAP may play a role in the selective protection afforded to females following early HI injury.

### 4.1. X-Linked IAPs and Brain Injury

Currently very little is known about the regulation of IAPs and XIAP in neonatal HI injury, though surprisingly, results from studies of XIAP knockout [93] and overexpression [94] have largely failed to report sex differences in degree of tissue damage following early HI injury. Further investigation revealed that these results may be due to compensatory changes in other IAP family members (i.e., upregulation of c-IAP1 and c-IAP2 [95, 96]), with XIAP remaining a probable source of protection for females. One specific study examined the long-term behavioral effects of neonatal HI following inhibition of XIAP in male and female rats [73]. Based on cumulative evidence of sex differences in apoptotic mechanisms, coupled with evidence of potential female protection via XIAP, this study utilized embelin—a small molecule inhibitor of XIAP. Embelin binds to the BIR3 domain (the biding site of caspase-9) on the XIAP protein molecule [91], thus preventing endogenous inhibition of apoptosis by XIAP. Treatment with embelin increased neuropathological damage and life-long behavioral deficits in HI females relative to vehicle-treated HI females, while no comparable effects were seen in males [73]. Thus, results demonstrate both the reliance on specific pathways of cell death between the sexes, as well as the importance of XIAP in the protection afforded to females following injury.

Clearly more research is needed on the role of IAPs in hypoxic-ischemic neuronal death, but the studies presented here emphasize the need for an improved understanding of innate mechanisms of protection in male and female neonates. Future studies will be needed to assess the potential interaction of hormonal exposure and genetic differences in sex chromosome gene expression within brain cells, since sex differences in response to early injury are almost certainly influenced by a combination of these factors.

## 5. Conclusions

Neonatal HI is a major cause of infant mortality and long-term neurologic morbidity in both preterm and term-injured populations. It is evident that the consequences of neonatal HI injury are severe, yet the difference in outcome experienced between the sexes is surprising. Male infants not only exhibit increased risk for HI, but also display greater behavioral and cognitive disruption following HI injury as compared to matched female counterparts. Animal studies utilizing induced neonatal HI suggest that this sex discrepancy may be modulated by (1) the presence of sex-specific hormones (e.g., testosterone), (2) sex differences in the preferred mechanisms of apoptosis, and/or (3) the protective effect of IAPs (which may be in greater quantity in female brain) on the caspase-dependent apoptotic pathway. Indeed, all three of these mechanisms may interact with each other, and sex differences in the effects of neonatal HI outcome likely reflect an interplay of both genetic and hormonal factors. One possible study to dissociate these interactive mechanisms could entail the use of a four-core genotype (FCG) mouse model (described in [97]), in which the Sry (testis determining) gene is deleted from the Y chromosome and inserted onto an autosome. A cross between this type of male and an XX female can then produce genetic females with insertion of the Y chromosome Sry gene modulating testicular development (thus leading to androgen exposure absent of all other Y chromosome genes), and XY males with knockout of the Sry gene (who develop as phenotypic females). Exploration of the consequences of neonatal HI in mice with Y genes but no testosterone, and testosterone but no other Y genes, could allow a more in-depth study of whether sex-based preference for apoptotic pathways may somehow be set by early androgen exposure, other genetic factors, or both.

 In closing, further studies of the influence of both genetic and hormonal factors relevant to neonatal HI could have important clinical implications. For example, the modulation of hormonal mechanisms leading to increased damage in males, modulation of apoptotic cascades, or modulation of IAPs may all represent target candidates for therapeutic intervention in neonates suffering HI brain injury. Further, studies looking at neonatal HI while manipulating testosterone receptors, estrogen receptors, and/or aromatase blockers could potentially dissociate or clarify the mechanism of action promoting injury. Moreover, it seems plausible that a lack of exposure to placental hormones due to premature birth could also be detrimental to the neurological development of premature infants (though no studies, of which we are aware, have determined such effects). Given the tremendous amount of research focusing on sex differences in adult stroke, we suggest that future research should similarly focus on sex differences in the consequences of neonatal HI. In fact, research in this area could yield beneficial sex-specific neuroprotectants, with far reaching implications for improved clinical practice and treatment.
